# Human in vitro reporter model of neuronal development and early differentiation processes

**DOI:** 10.1186/1471-2202-9-31

**Published:** 2008-02-29

**Authors:** Sebastien Couillard-Despres, Eike Quehl, Katrin Altendorfer, Claudia Karl, Sonja Ploetz, Ulrich Bogdahn, Juergen Winkler, Ludwig Aigner

**Affiliations:** 1 Department of Neurology, University of Regensburg, Universitätsstr. 84, 93053 Regensburg, Germany

## Abstract

**Background:**

During developmental and adult neurogenesis, doublecortin is an early neuronal marker expressed when neural stem cells assume a neuronal cell fate. To understand mechanisms involved in early processes of neuronal fate decision, we investigated cell lines for their capacity to induce expression of doublecortin upon neuronal differentiation and develop *in vitro *reporter models using doublecortin promoter sequences.

**Results:**

Among various cell lines investigated, the human teratocarcinoma cell line NTERA-2 was found to fulfill our criteria. Following induction of differentiation using retinoic acid treatment, we observed a 16-fold increase in doublecortin mRNA expression, as well as strong induction of doublecortin polypeptide expression. The acquisition of a neuronal precursor phenotype was also substantiated by the establishment of a multipolar neuronal morphology and expression of additional neuronal markers, such as Map2, βIII-tubulin and neuron-specific enolase. Moreover, stable transfection in NTERA-2 cells of reporter constructs encoding fluorescent or luminescent genes under the control of the doublecortin promoter allowed us to directly detect induction of neuronal differentiation in cell culture, such as following retinoic acid treatment or mouse Ngn2 transient overexpression.

**Conclusion:**

Induction of doublecortin expression in differentiating NTERA-2 cells suggests that these cells accurately recapitulate some of the very early events of neuronal determination. Hence, the use of reporter genes under the control of the doublecortin promoter in NTERA-2 cells will help us to investigate factors involved early in the course of neuronal differentiation processes. Moreover the ease to detect the induction of a neuronal program in this model will permit to perform high throughput screening for compounds acting on the early neuronal differentiation mechanisms.

## Background

Neurogenesis relies on a pool of proliferative neural stem cells differentiating into new neurons following induction of a neurogenic gene expression program. The whole maturation process from the multipotent neural stem cells into functional mature neurons involves several factors that orchestrate each step in an intricate continuum. A key event of neurogenesis is the neuronal commitment of multipotent neural stem cells, i.e. the time point at which cells lose their capacity to generate neurons as well as glia, and become restricted to the generation of new neurons.

Doublecortin (DCX) constitutes a marker for neuronal precursors throughout developmental and adult neurogenesis [1-4]. We demonstrated that DCX was expressed very early in newly generated neuronal precursors of the adult CNS [3]. Recently, a cortical subpopulation of multipotent DCX- NG2-expressing cells was reported in the adult CNS [5]. However, the identity of these cells and how they related to neurogenic processes has currently not been elucidated.

Although expression of DCX is well documented in neuronal precursors *in vivo *and in primary cell cultures, a cell line inducing sustained expression of DCX upon differentiation needed so far to be identified. The identification of such an *in vitro *model reproducing the early phases of neuronal determination would greatly accelerate the study of factors influencing neurogenesis. NTERA-2 cells have been previously reported to induce a complex neuronal differentiation program in response to retinoic acid (see for examples [6-8]). Based on the induction of DCX and DCX-driven reporter constructs upon differentiation, we further substantiated evidences establishing the NTERA-2 cell line as an appropriate and convenient *in vitro *model to study early steps of neuronal differentiation and factors acting on this process during development and in the adult CNS.

## Results

### Morphological changes in response to retinoic acid treatment

Five neuronal-like cell lines were tested for their capacity to mimic early events of neuronal determination and differentiation taking place during development or adult neurogenesis: D283 medulloblastoma (human medulloblastoma), DAOY (human medulloblastoma), NTERA-2 (human teratocarcinoma), PC-12 (rat pheochromacytoma), SK-N-SH (human teratocarcinoma). Additionally, HeLa cells (human adenocarcinoma) were chosen as non-neuronal control cell line. To differentiate cell lines towards a neuronal phenotype, retinoic acid (10 μM) was added to the cultures.

**Figure 1:**
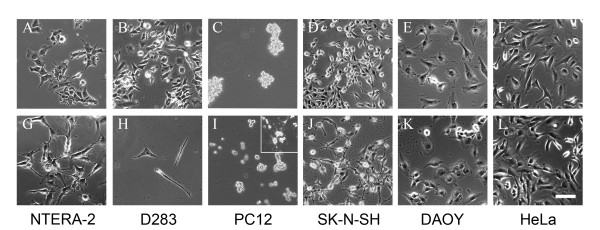
**Morphological changes in response to retinoic acid treatment**. Cell lines grown in standard conditions (A-F) or treated with retinoic acid (G-L). Only NTERA-2 cells (A *vs*. G) and D283 cells (B *vs*. H) showed a morphological response to retinoic acid. PC12 cells treated with NGF developed a complex network of cellular processes (I inset). Scale bar in L = 100 μm.

Figure 1 shows the morphological appearance of cell lines in response to retinoic acid treatment. Hence, after 28 days of differentiation, NTERA-2 cells morphology showed pronounced reorganization characterized by the formation of cell clusters and processes creating a complex cellular network (Fig 1A and 1G). In addition, D283 cell lines adopted a flattened morphology and showed increased cell death during differentiation treatment (Fig 1B and 1H). Finally, minor or no morphological responses could be detected for DAOY, HeLa, PC-12 or SK-N-SH cells following retinoic acid treatment.

PC-12 cells are known to undergo neuronal morphological differentiation in the presence of nerve growth factor (NGF). To confirm the potential of our PC-12 cultures to adopt a neuronal morphology upon differentiation, we maintained them four weeks in the presence of NGF (50 ng/ml). As previously described, PC12 cells underwent an extensive process outgrowth in response to NGF (Fig 1I inset).

### Induction of early neuronal marker expression in response to retinoic acid

**Figure 2:**
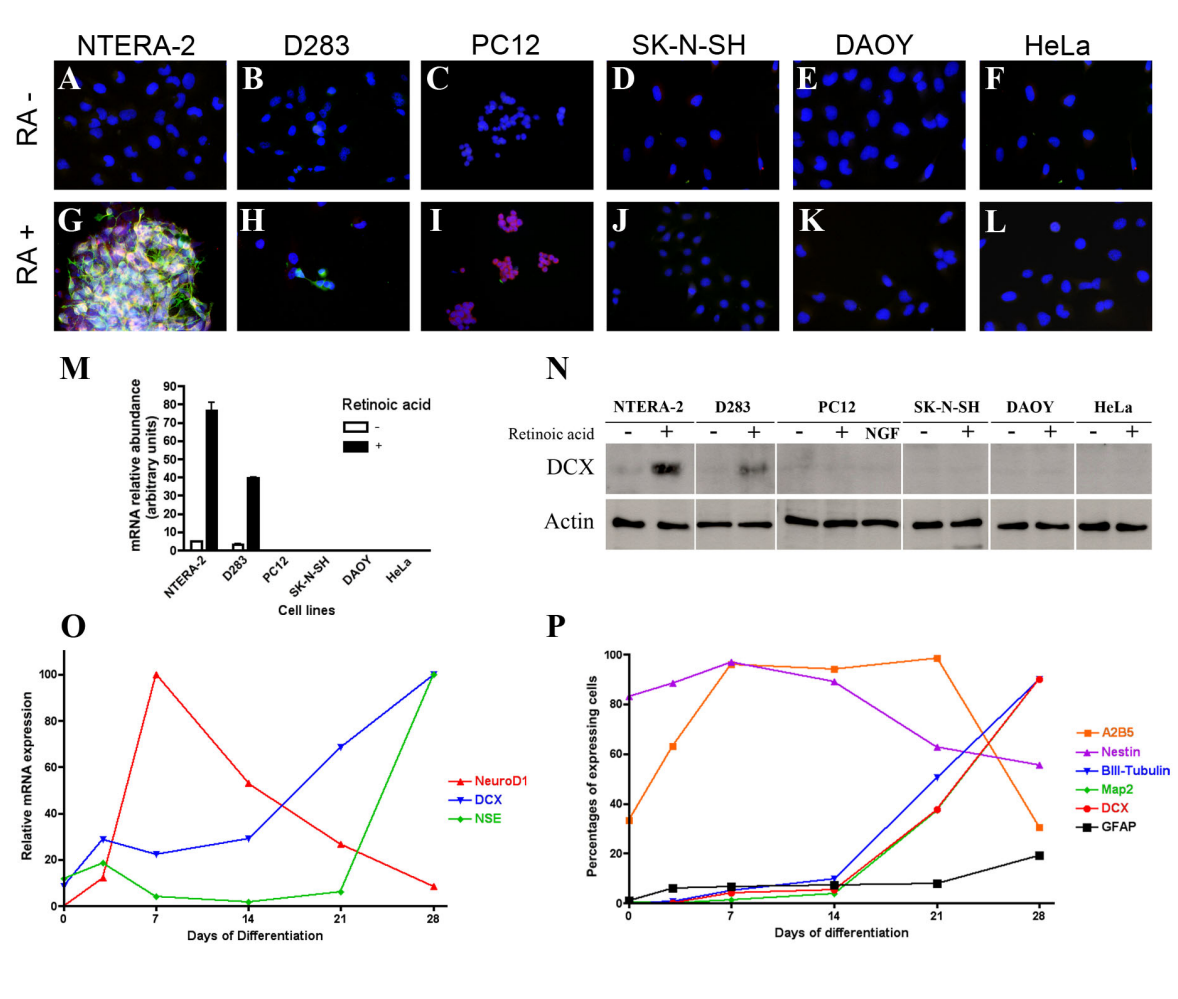
**Induction of DCX expression in response to retinoic acid treatment**. Cell lines grown in standard conditions did not express DCX (green) (A-F), whereas following retinoic acid treatment NTERA-2 cells (G) and to a lower extent D283 cells (H) expressed DCX. The presence of Map2 (red) could also be detected in most DCX-expressing NTERA-2. Strong induction of DCX mRNA expression (M) and protein expression (N) were also detected in NTERA-2 cultures and to a lower level in D283 cultures following retinoic acid treatment. Treatment of PC12 with retinoic acid or NGF did not result in an induction of DCX protein expression. O) In response to retinoic acid, differentiation of NTERA-2 cells resulted in the successive induction of NeuroD1, DCX and NSE mRNAs expression. P) Immunocytochemistry on retinoic acid-treated NTERA-2 cultures revealed an early induction of A2B5 expression, followed by the induction of the neuronal markers βIII-tubulin, DCX and Map2. GFAP-expressing cells remained a minor fraction within the cultures.

To examine if morphological changes observed in response to retinoic acid correlated with expression of genes induced during neuronal early differentiation, expression of DCX was assessed by immunocytochemistry. Figure 2(A–F) shows the absence of DCX expression in undifferentiated cells from the six cell lines investigated. Following differentiation however, robust expression of DCX could be detected in a large fraction of the NTERA-2 cells, especially those forming the cell clusters (Fig 2G). Expression could also be detected at a low level in differentiated D283 cells (Fig 2H). Moreover, most neuronal-differentiated NTERA-2 cells expressing DCX also induced expression of the neuronal-specific microtubule-binding protein Map2, hence revealing some degrees of neuronal maturation (Fig 2G). In contrast, only a few DCX-expressing differentiated D283 cells did express Map2. Finally, Map2 expression could be detected in differentiated PC12 cells and rarely in HeLa cells as well, however, DCX expression could never be detected in these cell lines.

### Induction of DCX expression in response to retinoic acid

Induction of DCX mRNA expression following retinoic acid treatment was further quantified in total RNA from each cell lines either in a proliferative state or following differentiation. In agreement with observation made by immunocytochemistry, undifferentiated cell lines did not express DCX, or did only in trace amounts (Fig 2M). On the other hand, following retinoic acid-induced differentiation, levels of DCX mRNA in NTERA-2 cells were increased approximately 16 times. Likewise, DCX mRNA levels were approximately 12 times more elevated in differentiated D283 cells then in the non-differentiated cells. The absolute DCX mRNA levels in D283 cells, however, were roughly half of those found in NTERA-2 cells. Finally, DCX mRNA expression was not detected in the other differentiated cell lines.

To examine the induction of DCX expression in respect to other neuronal genes, we followed the expression of NeuroD1, DCX and neuron-specific enolase (NSE) at various differentiation time points, i.e. proliferative state and following 3 days, 7 days, 14 days, 21 days and 28 days of retinoic acid treatment. As shown in figure 2O, retinoic acid treatment rapidly and transiently induced high levels of NeuroD1 expression, a neurogenic transcription factor [9]. As NeuroD1 expression declined between day 14 and 21 of differentiation, expression of DCX mRNA was induced and further increased until the last time point at 28 days. Finally, expression of NSE mRNA, an enzyme found in mature neurons [10], was induced only after the third week of differentiation. Hence, our differentiating NTERA-2 cultures expressed a key neurogenic factor, a neuronal precursor marker, and a mature neuronal marker in successive phases. This suggests that differentiation of NTERA-2 cells mimicked faithfully the various phases of the neuronal determination and differentiation program also induced in neural stem cells.

Western blot analysis substantiated the induction of DCX expression measured at the mRNA level. Whereas, very low levels of DCX protein expression could be measured in proliferating NTERA-2 cells, strong expression could be detected in differentiated NTERA-2 cells (Fig 2N). In addition, low levels of DCX protein expression could be detected in differentiated D283 cells (Fig 2N), in agreement with the lower abundance of DCX mRNA measured in these cells (Fig 2M). The presence of DCX protein could not be documented in the other cells lines, notwithstanding their differentiation status.

Expression of several neural markers in differentiating NTERA-2 cells was scrutinized in more details by immunocytochemistry at various time points. As shown in figure 2P, within the first 7 days of exposition to retinoic acid, the expression A2B5, a neural cell surface antigen, was induced in a large proportion of the cells, in agreement with previous report [8]. Expression of nestin, an intermediate filament expressed in neural stem cells, was found in a high percentage of NTERA-2 cells at all time points. Nestin expression, however, decreased slowly during the course of differentiation. In contrast, NG2, an antigen present on the cell surface of glial precursors, could not be detected in our NTERA-2 cultures at any time point.

Expression of neuronal markers became significant after 3 weeks of differentiation. Hence, expression of βIII-tubulin, DCX and Map2 were strongly induced and following 4 weeks of retinoic acid treatment, was found in more then 90% of the cells present in our NTERA-2 cultures. Expression of GFAP was detected in a minor fraction of the NTERA-2 cells at every time point. Finally, expression of NeuN, a marker found in more mature neurons, could not be detected using our differentiation paradigm.

### In vitro model of neuronal determination

This capacity of differentiating NTERA-2 cells to induce expression of the DCX gene was further exploited to establish an *in vitro *reporter system for neuronal early differentiation events. We recently reported that a genomic fragment of 3,5 kb of the human DCX gene drove expression of reporter genes in neuronal precursors and young neurons *in vitro *and *in vivo *[11,12]. We therefore generated stable NTERA-2 cell lines bearing the EGFP or the firefly luciferase reporter gene under the control of the DCX promoter sequences (NTERA-2^DCX-EGFP ^and NTERA-2^DCX-luci^). In parallel, we also generated transfected HeLa cell lines (HeLa^DCX-EGFP ^and HeLa^DCX-Luci^) as non-neuronal controls. Integration of the reporter construct was confirmed by PCR on the genomic DNA (data not shown).

**Figure 3:**
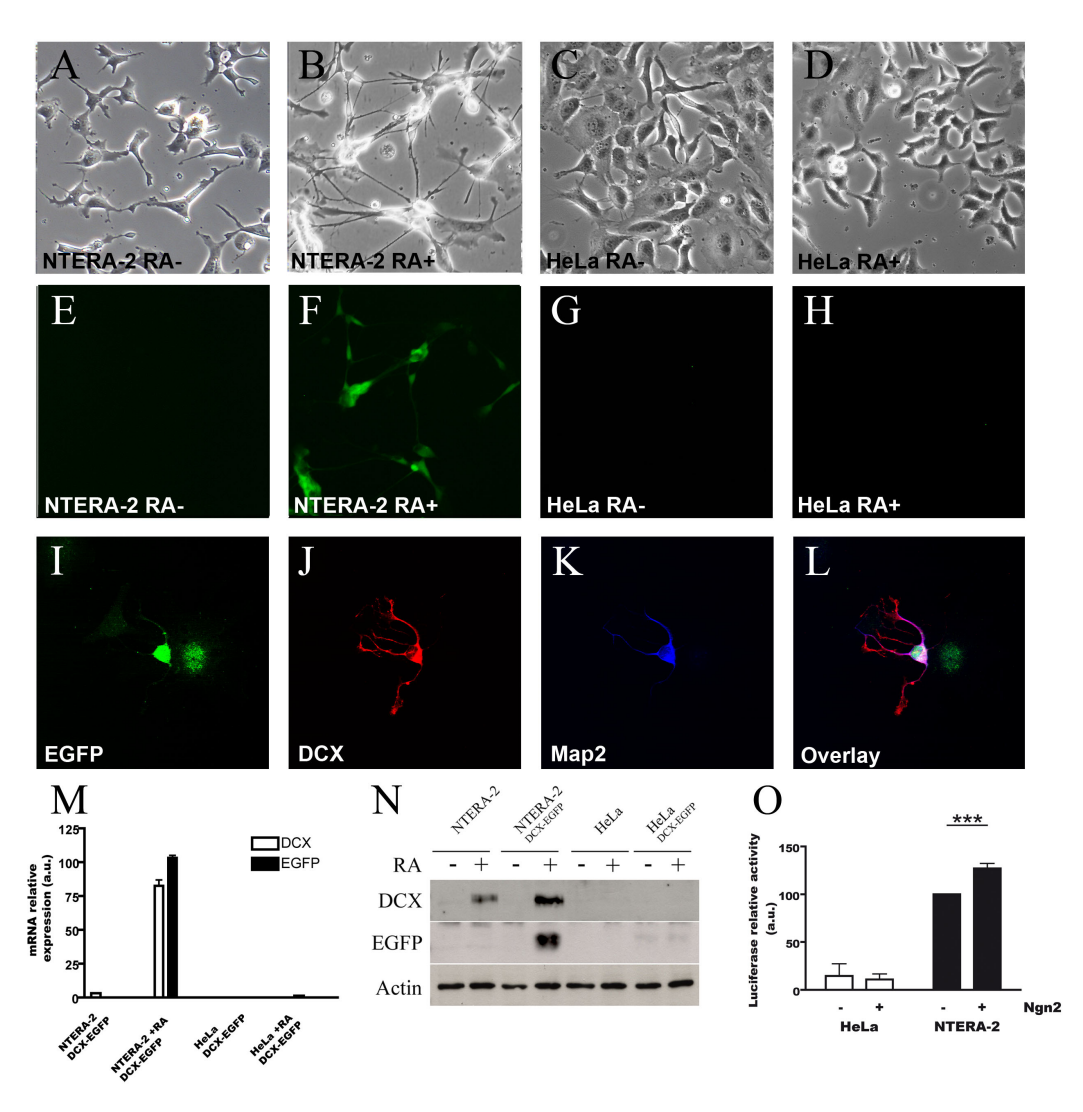
**Activation of DCX-promoter-reporter constructs in differentiating NTERA-2 cells**. Upon differentiation using retinoic acid, NTERA-2^DCX-EGFP ^clones (B and F), but not the HeLa^DCX-EGFP ^clones, could induce the expression of the DCX-promoter-EGFP reporter (A-H). NTERA-2^DCX-EGFP ^cells inducing the expression of EGFP upon retinoic acid treatment (I), also expressed DCX (J) and frequently Map2 (K). Parallel upregulation of the EGFP reporter and DCX mRNAs (M) and proteins (N) upon retinoic acid differentiation could also be detected in NTERA-2 clones, but not in HeLa clones. Induction of a neurogenic differentiation program following transient transfection of mouse Ngn2 also resulted in a significant induction of DCX-promoter-luciferase reporter in NTERA-2 clones, but not in HeLa clones (O).

Whereas undifferentiated NTERA-2^DCX-EGFP ^and HeLa^DCX-EGFP ^did not express the EGFP reporter, retinoic acid treatment strongly and specifically induced the expression of EGFP in the NTERA-2^DCX-EGFP ^cells but not in the HeLa^DCX-EGFP ^cells (Fig 3A–H). Immunocytochemistry further confirmed that EGFP-expressing cells also expressed DCX, and frequently Map2 as well (Fig 3I–L).

The induction of the reporter gene in differentiating NTERA-2 cells was examined at the mRNA and protein levels. In agreement with the strong induction of the endogenous DCX mRNA expression following differentiation of NTERA-2^DCX-EGFP ^cells, expression of the EGFP reporter mRNA was observed to be strongly induced (Fig 3M). In contrast, HeLa^DCX-EGFP ^did not upregulated the expression of the EGFP reporter in response to retinoic acid treatment. The potent induction of the reporter's expression in NTERA-2 cells could also be documented by western blotting (Fig 3N). Again, as expected from the mRNA measurements, expression of the reporter was not induced in HeLa^DCX-EGFP ^cells, underscoring the neuronal specificity of the reporter system.

Neurogenin 1 and neurogenin 2 (Ngn2) are transcription factors of the basic helix-loop-helix (bHLH) family expressed in the earliest steps of mammalian cortical neurogenesis. They initiate a cascade of gene activation leading to the expression of terminal neuronal differentiation genes [13,14]. It was recently reported that mouse Ngn2 could bind directly on the DCX promoter and activate it [15]. The capacity of our reporter system to respond to neurogenic factors was tested using a mouse Ngn2-encoding vector to transiently transfect the NTERA-2^DCX-luci ^cells. HeLa^DCX-Luci ^cell were used in parallel as a non-neuronal control. As shown in figure 3O, brief expression of mouse Ngn2 for as little as four days sufficed to activate DCX-promoter driven expression of the luciferase reporter gene in NTERA-2 cells, but not in HeLa cells.

## Discussion

Over the last few years, DCX became an established marker for neuronal determination and neurogenesis [4,12,16]. In differentiating neural stem cells, either *in vivo *or *in vitro*, expression of DCX can be detected upon induction of neuronal differentiation. To date however, no established neural cell line was shown to have the capacity to up-regulate DCX upon differentiation. In this report, we show that NTERA-2 cells in conjunction with DCX reporter constructs constitute an attractive model for the study of early phases of neuronal differentiation occurring during developmental and adult neurogenesis. Although, ultimate validation of factors acting on neuronal differentiation would need to be performed on primary cells, there was a need for such an *in vitro *system behaving as a simple neuronal differentiation model.

In contrast to other neural cell lines tested, such as PC12 cells, NTERA-2 and D283 cells could induce expression of DCX mRNA in response to retinoic acid exposure, a treatment known to induce a strong neurogenic response (for example Guan et al. [17]). At the protein level however, only differentiated NTERA-2 cells showed significant DCX expression. Expression of DCX in NTERA-2 cells was associated with the establishment of a neuronal morphology, as well as with the expression of other neuronal-specific markers, such as Map2 and NSE.

DCX expression occurs very early during the neuronal determination/differentiation process, possibly due to the direct binding of the bHLH transcription factors such as Ngn2 on the DCX promoter [15]. Hence, it appears that differentiating NTERA-2 cells can mimic neural stem cells in the early phase of neuronal differentiation. Supporting data also come from a recent gene profiling of NTERA-2 cells treated with retinoic acid. The latter revealed an expression pattern compatible with early neuronal phenotypes found in the posterior hindbrain and other posterior CNS regions [18]. Therefore, NTERA-2 cells appear to be well suited to be used as an *in vitro *model to study the early events of neuronal determination/differentiation and the factors influencing it.

In addition, we showed that genetically modified NTERA-2 cells bearing reporter genes under the control of the human DCX promoter constitute a simple and valuable tool to study factors acting on neuronal determination and early differentiation. We have previously demonstrated that DCX promoter-based expression vectors can drive expression of reporter genes in neuronal precursors and young neurons, both *in vitro *and *in vivo *[11,12]. The fact that reporter expression was not induced in HeLa cells further supports the evidence that expression of DCX promoter-based construct in NTERA-2 cells accurately reflects the induction of a complex genetic program similar to the early phase of neural stem cells differentiation into neurons, and not merely a *cis*-action on the DCX promoter.

## Conclusion

We could demonstrate in this report that expression of DCX and DCX promoter-driven reporters occurred in NTERA-2 cells upon induction of neuronal differentiation. Through this new and simple reporter system tool, it will be possible to analyze early events of neuronal determination and differentiation such as occurring during development and adult neurogenesis and identified new neurons directly *in statu nascendi*. Moreover, this culture model also provides a rapid screening method for molecules influencing the early process of neuronal differentiation.

## Methods

### Cell cultures

Cells were maintained in a humidified incubator at 37°C under an atmosphere containing 5% of CO_2_. HeLa, SK-N-SH, DAOY and D283 medulloblastoma cells were cultivated in MEM(Eagle) with Earle's BSS, supplemented with 10% (v/v) fetal calf serum, 2 mM L-glutamine, 1 mM sodium-bicarbonate, 0.1 mM non-essential amino acids, 100 U/ml Penicillin, 0.1 mg/ml Streptomycin. PC-12 were cultivated in RPMI 1640, supplemented with 10% horse serum, 5% (v/v) fetal calf serum, 100 U/ml Penicillin, 0.1 mg/ml Streptomycin, 2 mM L-glutamine. Finally, NTERA-2 cells were maintained in DMEM, supplemented with 10% (v/v) fetal calf serum, 4 mM L-glutamine, 4.5 g/l Glucose, 100 U/ml Penicillin, 0.1 mg/ml Streptomycin, 1.5 g/l sodium-bicarbonate.

### Cell differentiation

Cells were plated in a T75 culture flask at a density of 1.5 × 10^4 ^cells/cm^2 ^in their corresponding media. For the differentiation of D283 and PC-12 cell lines, the culture flasks were coated with poly-ornithine (250 μg/ml) to increase their adherence. One day after seeding, the differentiation was initiated according to a differentiation protocol adapted from [7]. Differentiation was induced by the addition of 10 μM of retinoic acid in the culture media. Cells were maintained under these condition for a period of 2 weeks, during which medium was replaced every 3 days. Thereafter, cells were further cultivated for 2 weeks in their designated medium in absence of retinoic acid. Retinoic acid treatment was previously reported to remodel the genetic program of PC12 cells [19]. Nevertheless, a standard differentiation protocol for the PC12 cells using 50 ng/ml nerve growth factor (NGF) in the culture medium was also applied to confirm their capacity to differentiate into neuron-like cells [20,21].

### Cell transfections

For stable transfection, cells were plated one day before transfection at a density of 1.5 × 10^5 ^cells/cm^2 ^in a T75 culture flask. Vectors encoding the EGFP reporter gene, or the firefly luciferase reporter gene, driven by the human DCX promoter [12] where transfected using Metafectene (Biontex Laboratories, Munich) according to manufacturer's guidelines. Selection of transfected cells began 3 days after transfection using Geneticin (250 μg/ml for HeLa and 300 μg/ml for NTERA-2). After 4 weeks under selection conditions, stable transfected single cells were FACS sorted into 96 wells/plate to establish clonal cultures. In the stably transfected clones, integration of reporter genes was confirmed by PCR on genomic DNA (EGFP-fow GCT GAC CCT GAA GTT CAT CTG, EGFP-rev GGA CTT GAA GAA GTC GTG CTG, Luciferase-fow TCA AAG AGG CGA ACT GTG TG and Luciferase-rev TTT TCC GTC ATC GTC TTT CC, beta-actin-fow AGC CAT GTA CGT AGC CAT CC, beta-actin-rev CTC TCA GCT GTG GTG GTG AA). Vectors used for transfection served as PCR control. Non-differentiated cells expressing the EGFP reporter gene constitutively, as a consequence of an insertion artifact for example, were excluded.

### Transcription factor transfection

To induce the neuronal differentiation, a vector encoding the complete mouse Ngn2-coding sequence, from ATG to the stop codon, flanked with a Myc tag was transfected using Metafectene (Biontex Laboratories, Munich). We used the bicistronic expression vector pIRES2-DsRed-Express (Clontech) to drive the expression of Ngn2 under the control of the CMV promoter. As control, we used the empty vector pIRES2-DsRed-Express, devoid of Ngn2. On the following day, transfected cell lines were plated in a 96 wells/plate at a density of 5 × 10^4 ^cells/well in triplicates in their respective culture media without retinoic acid. Four days after transfection, cells were processed to measure the firefly luciferase activity using the Luciferase Assay System (Promega). Luciferase activities were reported in arbitrary units, with the activities measured in NTERA-2 cells transfected with the empty vector representing the reference (100%).

### Expression analysis

Gene expression analysis was performed via quantitative RT-PCR on total cDNAs isolated from undifferentiated and differentiated cell cultures. RNAs were isolated using the RNeasy-Kit (Qiagen, Hilden) and cDNAs were synthesized using the RETROscript kit (Ambion, Austin, Texas, USA). Quantitative detection of EGFP transcription product was performed using a Rotor-Gene 3000 real-time PCR apparatus (Corbett Research, Mortlake, Sydney, Australia) and SYBR green master mix (Brilliant SYBR Green, Stratagen). Signals obtained for the EGFP were normalized on the levels of rRNA 15S (RETROscript kit, Ambion). Expression levels are represented as EGFP/rRNA 15S ratio.

For the analysis of neuronal gene expression at various time points of differentiation, total RNAs were extracted from cultures using Tri Reagent (Sigma) according to the manufacturer's protocol. Total RNAs were further purified using the SV Total RNA Isolation System (Promega). Reverse transcription was performed using the Reverse Transcription System (Promega) and controls for each sample were also generated by omitting the reverse transcriptase from the reaction. Real-time PCR was realized using SYBR Green QPCR Master Mix (Stratagen) and a Mx3005P thermocycler (Stratagen). Measurements were normalized on the β-actin mRNA levels and represented for each gene relative to its maximal expression. The following oligos were used: β-actin fow AGC CAT GTA CGT AGC CAT CC; β-actin rev CTC TCA GCT GTG GTG GTG AA; EGFP fow GCT GAC CCT GAA GTT CAT CTG; EGFP rev GGA CTT GAA GAA GTC GTG CTG; DCX fow GGA AGG GGA AAG CTA TGT CTG; DCX rev TTG CTG CTA GCC AAG GAC TG; NeuroD1 fow CCA AAA AGA AGA AGA TGA CTA AGG; NeuroD1 rev AGC TGT CCA TGG TAC CGT AA [8]; NSE fow ATC GCG CCA GCC CTC ATC AGC; NSE rev TTT TCC GTG TAG CCA GCC TTG TCG [8].

Protein expression was analyzed on homogenate derived from non-differentiated or differentiated cultures. Cells were lyzed in SUB buffer (0.5% w/v SDS, 8 M Urea, 2% v/v β-mercaptoethanol) and protein concentrations were determined using the Bradford method (Bradford Reagent, Sigma). Proteins homogenates (3 μg per lane) were separated on a 12.5% polyacrylamide SDS-PAGE and transferred on nitrocellulose membrane for western blotting. Membrane blocking, antibody dilution and washes were performed in the gelatin-western buffer (20 mM Tris-HCl pH 7.3, 0,9% w/v NaCl, 1% v/v gelatin from cold water fish (Sigma-Aldrich, Taufkirchen), 0,1% v/v Tween-20). Primary antibodies were applied on the membrane overnight at 4°C at the following concentration: goat anti-DCX C18 1:1000 (Santa Cruz, California, USA), goat anti-EGFP 1:1000 (Rockland, Gilbertsville, PA, USA) and rabbit anti-actin 1:5000 (Sigma-Aldrich, Taufkirchen). Peroxidase-conjugated donkey anti-goat (Sigma-Aldrich) and peroxidase-conjugated donkey anti-rabbit antibodies were applied on membranes for 2 hours at room temperature. Following extensive washes, immuno-complexes were revealed using ECL-Plus Western Blotting Detection System (Amersham-Pharmacia, Freiburg) and photographic emulsion (Hyperfilm, Amersham-Pharmacia).

### Immunocytochemistry

Cells were fixed for 30 minutes in phosphate-buffered 4% paraformaldehyde. Following washes, cells were blocked for 1 hour with gelatin-histology buffer (100 mM Tris-HCl pH 7.4, 150 mM NaCl, 1% bovine serum albumin, 0.2% gelatin from cold water fish (Sigma) and 0.1% Triton X100). Antibody dilutions and subsequent washes with also performed in this gelatin-histology buffer. Incubation with the primary antibodies was performed overnight at 4°C at the following dilutions: mouse anti-A2B5 1:200 (Chemicon), mouse anti-βIII-tubulin 1:500 (Promega), goat anti-Doublecortin C18 1:250 (Santa Cruz Biotechnology), rabbit anti-GFAP 1:1000 (Dako), mouse anti-Map2ab 1:250 (Sigma), rabbit anti-nestin 1:200 (Chemicon), mouse anti-NeuN 1:500 (Chemicon), rabbit anti-NG2 1:200 (Chemicon). Fluorochrome-conjugated donkey secondary antibodies diluted 1:500 were applied for 2 hours at room temperature (Alexa Fluor, Molecular Probes). Nuclear staining was obtained using 0.25 μg/ul of DAPI before mounting in Prolong Gold antifade reagent (Molecular Probes)

## Abbreviations

bHLH, basic helix-loop-helix; DCX, doublecortin; EGFP, enhanced green fluorescent protein; NGF, nerve growth factor; Ngn2, neurogenin 2; NSE, neuron-specific enolase; RA, retinoic acid.

## Authors' contributions

SCD Designed the study, perform reporter experiments and drafted the manuscript.

EQ Performed cell culture, expression and histological analysis.

KA Performed cell culture, expression and histological analysis.

CK Cloned the expression vectors.

SP Contributed to cloning, cell culture and expression analysis.

UB Contributed to the design of the study design and manuscript draft.

JW Contributed to the design of the study design and manuscript draft.

AL Contributed to the design of the study design and manuscript draft.

All authors read and approved the final manuscript.
